# Preformulation Studies of a Stable PTEN-PDZ Lipopeptide Able to Cross an *In Vitro* Blood-Brain-Barrier Model as a Potential Therapy for Alzheimer’s Disease

**DOI:** 10.1007/s11095-020-02915-8

**Published:** 2020-09-04

**Authors:** Aikaterini Lalatsa, Yujiao Sun, Jose Ignacio Gamboa, Shira Knafo

**Affiliations:** 1 Biomaterials, Bio-engineering, and Nanomedicine (BioN) Lab, Institute of Biomedical and Biomolecular Sciences, School of Pharmacy and Biomedical Sciences, University of Portsmouth, White Swan Road, Portsmouth, PO1 2DT UK; 2 Department of Organic Chemistry I, University of the Basque Country (UPV/EHU), 20018 Donostia - San Sebastian, Spain; 3 Biophysics Institute, CSIC-UPV/EHU, University of the Basque Country (UPV/EHU), 48940 Leioa, Spain; 4 Ikerbasque, Basque Foundation for Science, 48013 Bilbao, Spain; 5 Department of Physiology and Cell Biology, Faculty of Health Sciences, and The National Institute for Biotechnology in the Negev, Ben-Gurion University of the Negev, Beer-Sheva, Israel

**Keywords:** Alzheimer’s disease, blood-brain barrier, hCMEC/D3 human cerebral endothelial cells, PTEN-PDZ lipopeptides, stability

## Abstract

**Purpose:**

Amyloid β (Aβ) drives the accumulation of excess Phosphatase and Tensin Homolog Deleted on Chromosome 10 (PTEN) at synapses, inducing synaptic depression and perturbing memory. This recruitment of PTEN to synapses in response to Aβ drives its interaction with PSD95/Disc large/Zonula occludens-1 (PDZ) proteins and, indeed, we previously showed that an oligo lipopeptide (PTEN-PDZ) capable of blocking such PTEN:PDZ interactions rescues the synaptic and cognitive deficits in a mouse model of Alzheimer’s disease. Hence, the PTEN:PDZ interaction appears to be crucial for Aβ-induced synaptic and cognitive impairment. Here we have evaluated the feasibility of using PTEN-PDZ lipopeptides based on the human/mouse PTEN C-terminal sequence, testing their stability in biological fluids, their cytotoxicity, their ability to self-assemble and their *in vitro* blood-brain barrier (BBB) permeability. Myristoyl or Lauryl tails were added to the peptides to enhance their cell permeability.

**Methods:**

Lipopeptides self assembly was assessed using electron microscopy and the thioflavin T assay. Stability studies in mouse plasma (50%), intestinal washing, brain and liver homogenates as well as permeability studies across an all human 2D blood-brain barrier model prepared with human cerebral endothelial cells (hCMEC/D3) and human astrocytes (SC-1800) were undertaken.

**Results:**

The mouse lauryl peptide displayed enhanced overall stability in plasma, ensuring a longer half-life in circulation that meant there were larger amounts available for transport across the BBB (Papp_0-4h_: 6.28 ± 1.85 × 10^−6^ cm s^−1^).

**Conclusion:**

This increased availability, coupled to adequate BBB permeability, makes this peptide a good candidate for therapeutic parenteral (intravenous, intramuscular) administration and nose-to-brain delivery.

Graphical Abstract
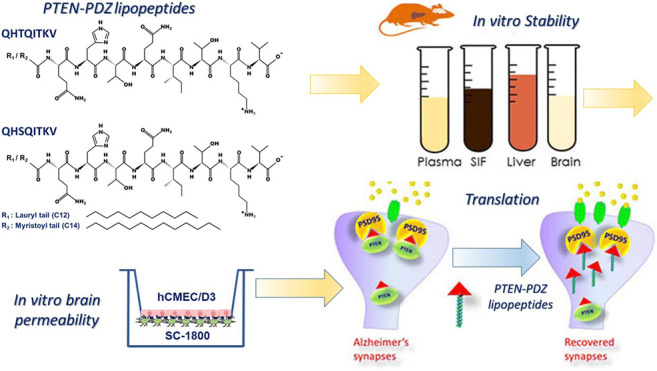

## Introduction

Peptides play essential roles in human physiology, acting as hormones, growth factors, neurotransmitters and, antibacterial agents, and their pharmacological characteristics define their strengths and weaknesses as potential drugs. In recent years, peptides have generated increasing interest in the scientific community, and they have been implemented in drug discovery programs by the pharmaceutical industry (1–4). Indeed, the role that peptide therapies can play in addressing unmet medical needs has been recognized, as has their potential to complement or surpass alternatives like small molecules and biological drugs (1). Like other protein-based therapeutic agents such as antibodies, peptides are generally very specific, and they are more likely to be sufficiently stable and permeable when used via non-invasive routes (5). In the case of Alzheimer’s disease (AD), the symptomatic therapies or disease-modifying agents available are on the whole unsatisfactory, and the number of drugs approved for human use has been limited by a high rate of failure of lead compounds in clinical trials (6).

Many approaches to treat AD have focused on lowering the levels of beta-amyloid (Aβ) in the brain but to date, most have been unsuccessful with the sole exception of aducanumab, an anti-amyloid antibody that reduces Aβ levels in the brain and that slows the rate of cognitive decline in individuals with mild or preclinical AD (6). However, the efficacy of this antibody in Phase III clinical trials has yet to be confirmed. Soluble Aβ assemblies directly alter synaptic plasticity by inhibiting long-term potentiation (LTP) and facilitating long-term depression (LTD) in hippocampal neurons (7), shifting the balance of synaptic plasticity towards a pathologically enhanced form of depression. We were recently encouraged by the discovery of a new approach to prevent memory loss in a mouse model of AD that does not target Aβ but rather, that focuses on preventing synaptic depression (7).

The lipid Phosphatase and Tensin Homolog Deleted on Chromosome 10 (PTEN) and phosphatidylinositol-3-kinase (PI3K) are essential regulators of excitatory synaptic structure and function, modulating the activity of phosphatidylinositol-(3,4,5)-triphosphate (PIP_3_) (8–11). The activity of PI3K in this pathway favors the formation of the PIP_3_ required for AMPA receptor (AMPAR) activity at synapses (12). By contrast, PTEN dephosphorylates PIP_3_ and favors AMPAR endocytosis during LTD. We found that PTEN is recruited to synapses during physiological LTD (13), and our latest data indicated that this process is enhanced in the presence of Aβ overload (7).

The participation of PTEN in LTD (13) strengthens the notion that Aβ acts, at least partially, by taking over physiological mechanisms of synaptic depression. Indeed, Aβ drives excess PTEN accumulation at synapses, and this induces synaptic depression and perturbs memory. Apart from PTEN, Aβ triggers the recruitment of other effectors of the PIP_3_ pathway into the postsynaptic compartment (e.g., Akt and GSK3β), which is accompanied by a downregulation of the PIP_3_ pathway in the dendritic spines (7,14). While the molecular mechanisms controlling this phenomenon are unknown, NMDAR (NMDA receptor) activation is required, as is an interaction between PTEN and synaptic proteins (7). Specifically, the recruitment of PTEN to synapses in response to Aβ drives its interaction with PSD95/Disc large/Zonula occludens-1 (PDZ) proteins. When this PTEN:PDZ interaction is prevented, as in PTEN^ΔPDZ^ mice in which PTEN lacks its C-terminal PDZ motif (-QITKV), normal basal synaptic transmission of neurons and LTP persists in the presence of Aβ (7). This finding suggests that the PTEN:PDZ interaction is crucial for Aβ-induced synaptic depression.

Based on this synaptic dysfunction triggered by Aβ, we suggest that therapeutic strategies that focus on inhibiting the PTEN:PDZ interactions may be potentially beneficial interventions in conditions of Aβ overload. Therefore, we designed a lipopeptide that corresponds to the last eight amino acids of rat/mouse PTEN, with an added myristic acid to augment its cell permeability (N-myristoyl-QHSQITKV - “Myr-PTEN-PDZ”). This compound interacts with several PDZ domains, of which the PDZ1 + 2 domains of PSD-95 are those most relevant to us, given that the PTEN-PSD-95 interaction is essential for the expression of LTD (13). Myr-PTEN-PDZ saturates the PDZ interaction sites of PTEN, thereby preventing PTEN from anchoring to PDZ proteins and impeding LTD. The inability of synapses to expresses LTD in the presence of Myr-PTEN-PDZ renders them resistant to Aβ-induced synaptic depression (7). Following direct administration of the Myr-PTEN-PDZ peptide into the cerebral ventricles of mice, the synaptic activity usually associated with Aβ overload and cognitive impairment was reversed (7). Hence, PTEN-PDZ peptides may represent pharmacological tools that, by saturating PTEN:PDZ interaction sites, effectively protect synapses from the deleterious effects of Aβ, preventing cognitive deterioration.

These discoveries provide us with a new mechanism-based therapeutic target to counteract signaling downstream of Aβ. As such, novel pharmacological interventions can be directed at modulating the interactions of PTEN with PDZ proteins, thereby preventing pathological LTD. We hypothesize that PTEN-PDZ peptides may impede or delay the onset of memory defects in the early phases of AD. Therefore, we used our mouse Myr-PTEN-PDZ as a prototype to develop other similar lipopeptides. Specifically, we replaced the myristoyl group with a lauryl group for both the corresponding mouse/rat and human sequences (N-Lauryl-QHSQITKV and N-Lauryl-QHTQITKV, respectively). Lipidized peptides are more stable in biological fluids (7,15) and they display better permeability across biological membranes like the blood-brain barrier (BBB) (2,5), the intestinal mucosa (3,16,17) and the nasal mucosa (18). Here we assess the stability of the lipopeptides developed in biological fluids relevant to parenteral routes (50% *v*/*v* plasma, 50% *w*/*v* liver, and 50% *w*/*v* brain homogenates), as well as in a simulated intestinal fluid (SIF) that is relevant to non-invasive routes. We also analyzed the cytotoxicity of the lipopeptides towards human immortalised cerebral endothelial capillary cells and their permeability across a human 2D BBB model, with a view to identifying PTEN-PDZ lipopeptides ideal for preclinical development and administration via a minimally invasive route. Accordingly, N-lauryl-QHSQITKV (mouse Lau-PTEN-PDZ) appears to be the leading candidate for further development as AD therapies administered via parenteral routes. These results represent the first and the critical step in the discovery, design, and subsequent development of novel therapeutics to combat memory loss in AD.

## Materials and Methods

### Materials

**Fig. 1:**
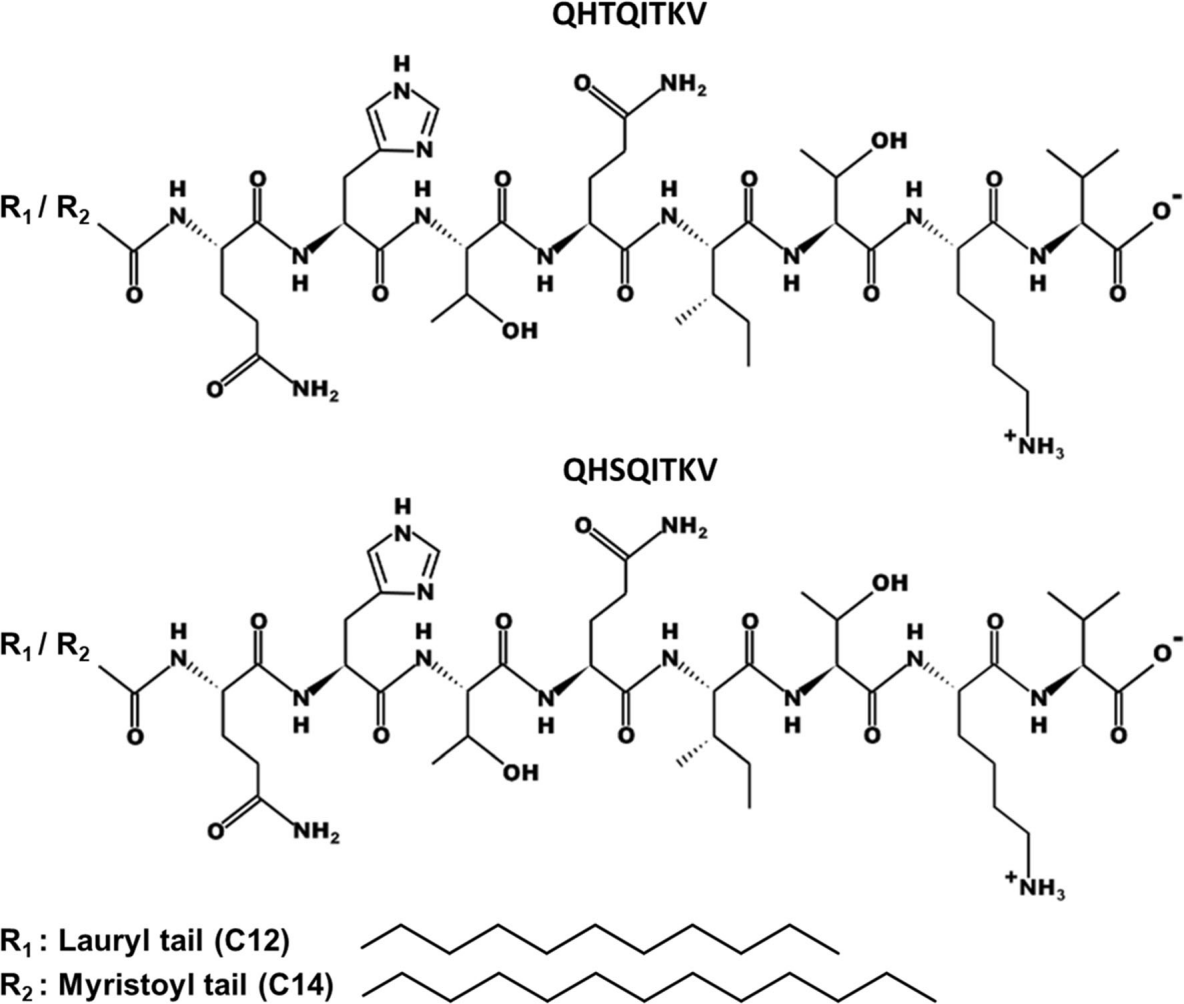
A. Chemical structures of the PTEN-PDZ lipopeptides.

Different peptides were synthesized by CASLO Laboratory ApS (Lyngby, Denmark), ≥98% pure by HPLC (Fig. 1): N-Lauryl-QHSQITKV (mouse Lau-PTEN-PDZ); N-Lauryl-QHTQITKV (human Lau-PTEN-PDZ); N-myristoyl-QHSQITKV (mouse Myr-PTEN-PDZ), N-myristoyl-QHTQITKV (human Myr-PTEN-PDZ). Other reagents were obtained from Sigma Aldrich (Gillingham, UK): diazepam (>98%), fluorescein isothiocyanate dextran (FITC-Dextran, 3-5KDa, ≥90%), human serum (heat-inactivated from human male AB plasma, sterile-filtered, USA origin), human fibronectin, Bradford reagent, bovine serum albumin (>98%, BSA), thioflavin T, and HPLC grade solvents (acetonitrile, methanol). Fisher Scientific (UK) supplied the BD Biosciences K3E EDTA vacutainers (evacuated, sterile, spray-coated with 3.6 mg of tripotassium ethylenediaminetetraacetic acid [EDTA], 3 × 75 mm medical-grade PET tubes), dimethylsulfoxide (99.5%, DMSO, molecular biology grade), trifluoroacetic acid (TFA, HPLC grade), sodium chloride (ACS grade), sterile-filtered Hank’s Balanced Salt Solution (HBSS without calcium, magnesium, and phenol red) and sterile-filtered Phosphate Buffered Saline (PBS, without calcium and magnesium, pH 7.4). Human cerebral microvascular endothelial cells (hCMEC/D3, passage number 32–40), immortalized with a human telomerase reverse transcriptase catalytic subunit, and a Simian vacuolating virus 40 (hTERT/SV40) cells were obtained from Dr. Pierre-Olivier Courard (Institute Cochin, INSERM, Paris). The hCMEC/D3 cells were cultured in endothelial basal medium-2 (EBM-2) supplemented with the EGM-2 Bullet Kit (Lonza Ltd., Slough, UK). SC-1800 astrocytes (ScienCells research laboratories, Carlsbad, CA, USA) were grown in complete astrocyte basal media (ABM™, CC-3187: Lonza Ltd., Slough, UK) and AGM™ (CC-4123) supplemented with SingleQuots™ (Lonza Ltd., Slough, UK).

### *In Vitro* Stability Studies in Mouse Plasma, Liver and Brain Homogenates

CD-1 male mice (7 weeks old: Charles River Laboratories) were sacrificed with CO_2_, and blood was collected in K3E EDTA spray-coated vacutainers and kept on ice prior to centrifugation at 3000 rpm for 15 min at 4°C (Heraeus Multifuge 3sf). Mouse plasma samples were stored at −80°C before use in stability studies. Study was approved by the University of Portsmouth Animal Welfare and Ethical Review Body (UoP AWERB body, 817).

For testing, the plasma was diluted (1:1) in PBS without phenol and pre-warmed to 37°C. Peptides stocks (5 mM in DMSO) were prepared and maintained at −20°C before performing the stability studies. Peptide stock solutions (120 μL) were added to pre-warmed diluted plasma (50% *v*/*v*, 1080 μL) and maintained at 37°C with agitation (100 rpm: Grant Aqua Waterbath, Royston, UK). Samples (80 μL) were taken at 0, 3, 6,9, 15, 30, 45, 60, 90, 120, 180, 240, 360 and 1440 min, diluted with ice-cold acetonitrile (80 μL), vortexed and left in −80°C at least overnight to favor maximal protein precipitation. The samples were then vortexed again for 15 min (SciQuip microplate shaker, Shropshire, UK), centrifuged in a Thermo Sci Jouan B4i Centrifuge (Hemel Hampstead, UK) and the supernatant was transferred to an HPLC vial (200 μL).

The samples were analyzed at 40°C on a Phenomenex Lichrosorb RP8 C8 column (4.0 mm × 250 mm, 5 μm), using the gradient elution method on an Agilent HPLC system (Agilent Technologies, Cheadle, UK) equipped with an 1100 series degasser, quaternary pump, autosampler, and column heater, and attached to an Agilent 1200 series PDA detector and a 1250 series fluorescent detector. The flow rate was set at 1.2 mL min^-1,^ and the injection volume was set at 40 μL, while detection was performed at 220 nm. The mobile phase was a mixture of mobile phase A (0.1% TFA in water) and phase B (0.08% TFA in acetonitrile), and in the first 5 min, the mobile phase was an 8:2 ratio of A:B (*v*/*v*) and from 5 to 15 min the percentage of B was raised from 8:2 to 1:9 (*v*/*v*). Linear calibration curves were constructed for both peptides between 0.1–100 μg.mL^−1^. The retention time for the human Lau–PTEN-PDZ, mouse Lau–PTEN-PDZ, human Myr–PTEN-PDZ, and mouse Myr–PTEN-PDZ was 12.03, 11.60, 13.68, and 13.91, respectively.

The liver and brain of the mice were excised from black CD-1 male mice (7 weeks-old: Charles River Laboratories) sacrificed with CO_2_, and the tissue was frozen immediately with liquid nitrogen prior to storage at −80°C. The liver and brain tissue were thawed and homogenized on ice in PBS (2 mL/g of tissue) using a glass 3 mL tissue homogenizer (20 strokes per sample), and the liver and brain homogenates were centrifuged (Heraeus Multifuge 3sf, Fisher Scientific, Basingstoke, UK) at 4300 g for 60 min at 4°C and at 3000 g for 20 min at 4°C, respectively. The supernatant was stored at −80°C, diluted 1:1 (*v*/*v*) with PBS (1x, pH 7.4), and used in stability studies. Peptides stocks (5 mM in DMSO) were prepared and maintained at −20°C prior to performing stability studies. These stock solutions (120 μL) were added to the diluted pre-warmed (37°C) liver or brain homogenate (50% *v*/*v*, 1080 μL) with agitation (100 rpm: Grant Aqua Waterbath), and samples (80 μL) were taken at 0, 5, 10, 15, 30, 45, 60, 90, 120, 180, 240, 360 and 1440 min, and diluted with ice-cold acetonitrile (80 μL). Samples were vortexed and left at −80°C at least overnight to allow for maximum protein precipitation, the samples were vortexed again for 15 min, centrifuged and the supernatant was analyzed by HPLC as described above.

The total protein concentration in the homogenates was determined using the Bradford protein assay (7,15) and BSA as a standard. Ten stock solutions of BSA were prepared in PBS between 0.1–1 mg mL^−1^, and 40 μL of each of the diluted stocks were added to 1.2 mL of Bradford reagent and incubated at room temperature for 5 min. The absorbance was recorded at 595 nm using a Multiskan GO spectrophotometer and the SkanIt software (Thermo Scientific, Paisley, UK). A linear calibration curve was obtained in this range that was used to extrapolate the protein content of 1:100 *v*/*v* stock solutions of 50% mouse plasma, liver, and brain homogenates diluted in PBS.

### *In Vitro* Stability Studies in Simulated Intestinal Fluid (SIF)

The duodenum and ileum were excised from 10 non-fasted CD-1 male mice (7 weeks-old: Charles River Laboratories) that were sacrificed with CO_2_. Each duodenum and ileum were washed with 6 ml of phosphate buffer (50 mM, pH 6.63), and all the washes were pooled prior to centrifugation at 4000 rpm for 15 min at 4°C (Heraeus Multifuge 3sf) (7,15). The intestinal wash was removed and frozen at −80°C prior to using it in stability studies without further dilution. Peptides stocks (5 mM in DMSO) were prepared and maintained at −20°C prior to performing stability studies. A stock peptide solution (120 μL) was added to the diluted pre-warmed (37°C) intestinal wash (1080 μL) with agitation at 100 rpm (Grant Aqua Waterbath), and samples (80 μL) were taken at 0, 15, 30, 45, 60, 90, 120, 180, 240, 360 and 1440 min, and diluted with ice-cold glacial acetic acid (17.5 M): acetonitrile (5:95 *v*/*v*, 80 μL) to quench the enzyme activity. Samples were vortexed and left in −80°C at least overnight to maximize protein precipitation, and after vortexing again for 15 min and centrifuging, the supernatant was analyzed by HPLC as described above.

### Critical Aggregation Concentration Studies

Thioflavin T, a probe that possesses freely rotatable dimethylaniline and benzothiazole ring around a shared bond, is used to assess the self-assembly of amphiphilic peptides in aqueous media. When immobilized in fibrils or aggregates, this probe maintains an excited state, resulting in an increase in fluorescence. Peptide stock solutions (0.1–40,000 μM) were prepared in DMSO and diluted 100-fold in PBS to yield solutions ranging from 0.001–400 μM. Diluted stocks (20 μL) were added to freshly prepared thioflavin T (50 μM, 80 μL) and incubated for 5 min at room temperature, protected from the light. The fluorescence of the solutions was then measured at λex 450 nm and λem 482 nm in black 96-well plates on a Synergy H1 microplate reader and with Gen 5 software (BioteK, Vermont, USA). The critical aggregation concentration (CAC) was calculated using the tangent method (19).

### Morphology of the Aqueous Dispersion of Lipopeptides Using Transmission Electron Microscopy (TEM)

TEM was used to study the morphology of the amphiphiles. Peptide stock solutions (40,000 μM) were prepared in DMSO and diluted 100-fold in PBS (Gibco 10,010–31, Lot:1930056) to yield 400 μM solutions. These solutions were diluted 1:5 in deionized water, and a drop of the diluted solution was placed on the coated side of a copper Formvar/Carbon coated grid (F196/100 3.05 mm, 300 mesh, TAAB, UK) and allowed to dry for 5 min. The sample was then negatively stained with a drop of 2% *w*/*v* uranyl acetate aqueous solution for 30 s. The grid was blotted with Whatman No1 filter paper and allowed to dry at room temperature. Images were analyzed on a JEM-1400 electron microscope (Jeol, Herts, UK), and the images were acquired with an AMT digital camera (Advanced Microscopy Techniques, Suffolk, UK) (20).

### Metabolic Assays in hCMEC/D3 Cells

HCMEC/D3 (passage number 32–40) cultured in EBM-2 (Lonza) supplemented with the EGM-2 Bullet Kit™ (Lonza), and 2% *v*/*v* human serum (Sigma H3667, Batch: SLBW2276) were used to assess the cytotoxicity of lipidized peptides. The cells were subjected to routine mycoplasma testing and authenticated in house. The cells were seeded at 46,875 cells/cm^2^ in complete medium, and allowed to attach overnight, refreshing the medium to treat the cells with peptide solutions. The peptide solutions were prepared from 100-fold DMSO stocks at a final concentration between 1 and 400 μM in complete medium to ensure a final DMSO concentration of 0.5%, and 200 μL of the diluted stock was added to each well. The metabolic activity of the cells was measured after 4 and 24 h using the MTT (3-(4,5)-dimethylthiazol-2-yl)-2,5-diphenyltetrazolium bromide) assay. At the time points specified, the MTT solution was added to each well (20 μL at 5 mg.mL^−1^ in PBS), and the cells were incubated for 4 h at 37°C. Subsequently, DMSO (100 μL) was added to dissolve the formazan crystals, and the absorbance was measured at 570 and 690 nm on a Multiskan Go microplate spectrophotometer, analyzing the data using the SkanIt software (Thermo Scientific, Paisley, UK). Cell metabolic activity was calculated by subtracting the values at 690 nm from those at 570 nm to remove the background and dividing the values by the control to express this value as a percentage (%) of the control (0.5% DMSO).

1 $$ \mathrm{Cell}\ \mathrm{Metabolic}\ \mathrm{Activity}\ \left(\%\right)=\frac{\left({\mathrm{Abs}}_{570\ \mathrm{nm}\ \mathrm{Sample}}-{\mathrm{Abs}}_{690\ \mathrm{nm}\ \mathrm{Sample}}\right)\times 100}{\left({\mathrm{Abs}}_{570\ \mathrm{nm}\ \mathrm{Control}}-{\mathrm{Abs}}_{690\ \mathrm{nm}\ \mathrm{Control}}\right)} $$

IC_50_ values were calculated using Graphpad Prism 8.0 (San Diego, USA) and fitted using a non-linear regression model ([Inhibitor] vs. response (three parameters)).

### Permeability Studies across an *In Vitro* 2D Human Blood-Brain Barrier (BBB) Model

HCMEC/D3 were cultured in EBM-2 (Lonza) supplemented with the EGM-2 Bullet Kit™ (Lonza) and 2% human serum (*v*/*v*: Sigma H3667, Batch SLBW2276). A static *in vitro* co-culture model was set up in 24-well Transwell polycarbonate membranes (3 μm pore size, 0.33 cm^2^ area: Corning Costar, Deeside, UK), the abluminal side coated for 1 h at room temperature with fibronectin from human plasma (5 μg/cm^2^ in HBSS). SC-1800 astrocytes (25,000 cells in 100 μL) were seeded onto the abluminal side in complete ABM (ABM™ Basal Medium, CC-3187, and AGM™ SingleQuots™ Supplements, CC-4123: Lonza) for astrocyte growth, and they were allowed to attach for 2 h at room temperature. The membranes were then flipped in the 24-well plates, and the SC-1800 cells were grown for 3 days in complete ABM media (600 μL and 200 μL on the luminal and abluminal side of the Transwell respectively). After 3 days in culture, the luminal side of the Transwell membranes was coated for 1 h at 37°C with fibronectin from human plasma (5 μg/cm^2^ in HBSS). HCMEC/D3 were cultured as above and seeded onto the luminal side of the membrane in 200 μL of complete EBM-2 (75,000 cells per Transwell), and then co-cultured with the astrocytes for 5 days. The medium on both sides of the Transwell was changed daily, adding 600 μL of complete ABM added to the abluminal side and 200 μL of complete EBM-2 on the luminal side of the Transwell.

To evaluate the integrity of the barrier, the trans-epithelial electrical resistance (TEER) was measured using an EVOM volt ohmmeter (World Precision Instruments, Berlin, Germany). All Transwells in a 24-well plate were equilibrated at room temperature while the EVOM electrodes were equilibrated in PBS (0.01 M, pH 7.4), giving a TEER of ~0 Ω.cm^2^. Electrodes were inserted into the wells at a 90^o^ angle, and the cell resistance was recorded in ohms (Ω). The resistance of a new membrane immersed in PBS was recorded to obtain the real resistance of the co-culture based on the following equation:

2 $$ \mathrm{TEER}=\left(\mathrm{TEER}\ \mathrm{sample}\hbox{--} \mathrm{TEER}\ \mathrm{blank}\right)\ \mathrm{x}\ \mathrm{Transwell}\ \mathrm{Surface}\ \mathrm{Area} $$

where the TEER of the blank Transwell was subtracted from the TEER of the co-culture and multiplied by the surface area of the membrane (0.33 cm^2^), giving the final TEER in Ω.cm^2^.

After 5 days and when the TEER value reached the maximum resistance (25 ± 1.63 Ω.cm^2^) due to tight junction maturation, the Transwell inserts were washed with PBS (no phenol red) and immersed in a fresh buffer (600 μL) placed on the abluminal side. The peptides dissolved in DMSO were diluted 200-fold in PBS and added to the luminal side of the Transwells (100 μM, 150 μL). Diazepam and FITC-dextran (3–5 kDa) were used as controls for the permeability studies. Diazepam is a lipophilic drug known to cross the BBB trans-cellularly (16), while FITC-dextran is known to cross the BBB in a luminal to abluminal direction paracellularly (21). Diazepam (150 μL, 50 μg mL^−1^ in PBS) and FITC-dextran (150 μL, 500 μg mL^−1^ in PBS) solutions were added to the luminal side of the Transwell, and the co-cultures were incubated at 37°C with agitation (150 rpm: Heidolph Titramax 1000, Heidorph, Schwabach, Germany). Samples (100 μL) were collected from the abluminal side at specific time points and replaced with an equal volume of fresh PBS. The samples were stored at −80°C for further analysis.

Peptides were analyzed by HPLC, as described above. All diazepam samples were analyzed on a Phenomenex Hypersil C18 CN column (150 × 4.5 mm, 5 μm) and eluted isocratically using an Agilent 1100 series RP-HPLC apparatus equipped with a quaternary pump, autosampler, and photodiode array detector (Agilent Technologies, Cheadle, UK). The mobile phase was acetonitrile:methanol: phosphate buffer (20 mM, pH 2.37) at a ratio of 27:10:63 *v*/*v*. Diazepam was eluted at a flow rate of 1.2 mL min^−1^ at room temperature, and the injection volume was set at 40 μL, detecting the samples at 230 nm and analyzing the results with OpenLAB software (Agilent Technologies, Cheadle, UK). The retention time was 2.75 min, and a calibration curve of diazepam in the mobile phase was prepared from 0.025–100 μg mL^−1^ to extrapolate the concentration of diazepam in the samples. The fluorescence of FITC-dextran samples was analyzed on a POLARstar Omega Plate Reader spectrophotometer (BMG LABTECH, Ortenberg, Germany), measured at λex 485 and λem 520 nm in black 96-well plates. A calibration curve of FITC-dextran in PBS was prepared from 0.25–500 μg mL^−1^ to quantify the FITC dextran concentrations.

The permeability coefficient (Papp) was determined using the equation below:

3 $$ {\mathrm{P}}_{\mathrm{app}}=\frac{\Delta {\mathrm{Q}}_{\mathrm{r}}}{\Delta_{\mathrm{t}}}\times \frac{{\mathrm{V}}_{\mathrm{r}}}{\mathrm{A}\times {\mathrm{C}}_0\times 60} $$

where ΔQr/Δ_t_ is the amount of compound in basal compartment (μg mL^−1^) as a function of time (minutes), Vr is the volume on the abluminal side (mL), A is the surface area of the Transwell membrane, Co is the concentration of drug on the luminal side, and 60 is the conversion factor from minutes to seconds. Two experiments were carried out due to the number of replicates possible per Transwell plate. Diazepam was included in both experiments as a positive control.

## Results

We designed four “PTEN-PDZ” peptides based on the last eight amino acids of the mouse/human PTEN, adding fatty acids (myristoyl or lauryl groups) to augment their cell permeability and stability. As part of our preclinical assessment of these four lipidized synthetic peptides, we evaluated their plasma, brain, liver, and gastrointestinal stability, their ability to self-assemble in aqueous media, and their toxicity in human immortalised cerebral endothelial cells, as well as their BBB permeability. This information was used to consider the most likely intended route of administration and to help select one of the synthetic lipidized peptide analogs to use in further preclinical development.

### The Sequence and Fatty Acid Group Determine the Stability

**Fig. 2:**
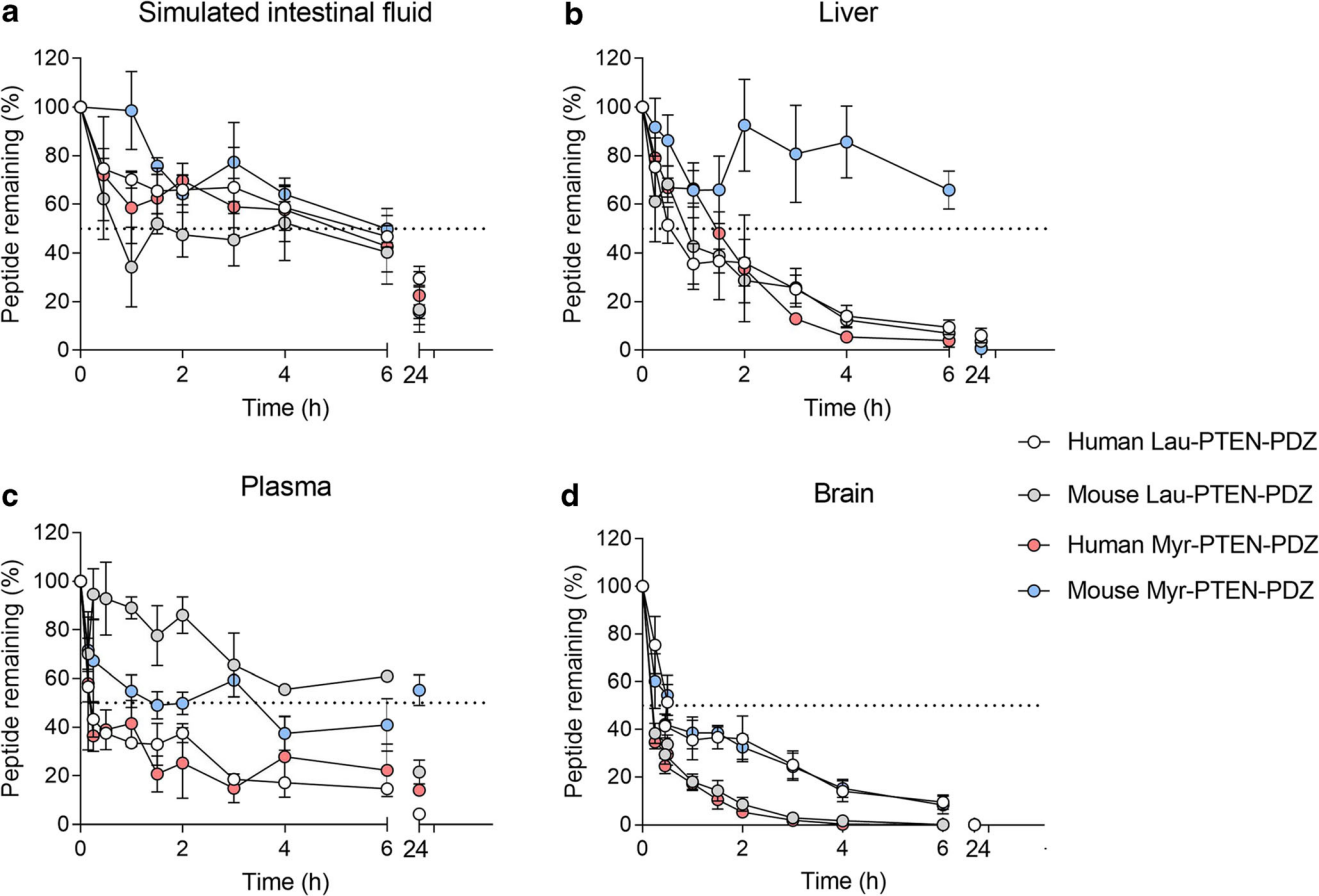
The proportion of the peptide remaining after incubation in 50% *v*/*v* plasma, 50% *w*/*v* brain homogenate, 50% *w*/*v* liver homogenate, or simulated intestinal fluid (SIF). The data are presented as the means ± SEM (*n* = 3). The protein content was quantified using the Bradford assay and the protein content of 50% *v*/*v* plasma, 50% *w*/*v* brain homogenate, 50% *w*/*v* liver homogenates, and SIF was 8.71 ± 0.30, 8.01 ± 0.75, 60.42 ± 6.81 and 1.05 ± 0.21 mg mL^−1^ respectively.

Ideally, an oral formulation for AD would revolutionize current treatment options. The first hurdle that a peptide faces following oral delivery are the high salt and acidic environment of the stomach, which, when combined with the action of pepsin, can denature the peptide. As little is known about the stability of the peptides understudy in the gastrointestinal tract, we assessed their stability in a soluble fraction of the digestive enzymes present in the upper gastrointestinal tract, where the peptide must be released for absorption and where it is likely to be attacked by proteolytic enzymes (15). This soluble fraction contains pancreatic proteases like trypsin and chymotrypsin, carboxypeptidases and aminopeptidases that can hydrolyze peptides to di- or tripeptides (5). Our stability studies in a simulated intestinal fluid (SIF) indicate that the prototypic mouse Myr-PTEN-PDZ lipopeptide shows the best stability (Fig. 2a and Table I) and if oral delivery is considered, this peptide will have an advantage in eliciting the largest window for oral absorption, thereby resulting in the highest oral bioavailability. Nevertheless, the industrial processes currently available for enteric coating can easily overcome the hurdle if peptides are unstable in the upper gastrointestinal tract (5).

**Table I:** Summary of the half-livesof the peptides

Peptide	Equation fitted	k (h^−1^)	t_1/2_ (h) / t_1/2_ (min)	r^2^
Simulated intestinal fluid				
Human Lau-PTEN-PDZ	One phase decay, Least square fit	0.23	2.96 / 177.96	0.22
Mouse Lau- PTEN-PDZ	One phase decay, Least square fit	2.34	0.30 / 17.77	0.47
Human Myr-PTEN-PDZ	One phase decay, Least square fit	0.25	Could not be calculated due to fitting (deviation)	0.12
Mouse Myr-PTEN-PDZ	One phase decay, Least square fit	0.15	4.52/ 271.02	0.74
50% *w*/*v* Liver Homogenate				
Human Lau-PTEN-PDZ	One phase decay, Least square fit	1.15	0.60 / 36.28	0.79
Mouse Lau- PTEN-PDZ	One phase decay, Least square fit	0.73	0.95 / 57.14	0.75
Human Myr-PTEN-PDZ	One phase decay, Least square fit	0.04	18.5 / 1110	0.49
	Two phase decay	3.05 (fast), 0.0005 (slow)	0.23 (fast) & 1510 (slow) / 13.63 (fast) & 90,600 (slow)	0.54
Mouse Myr-PTEN-PDZ	One phase decay, Least square fit	0.51	1.36/ 81.66	0.85
50% *v*/*v* Plasma (Mouse)				
Human Lau-PTEN-PDZ	One phase decay, Least square fit	4.60	0.15 / 9.05	0.83
Mouse Lau- PTEN-PDZ	One phase decay, Least square fit	0.12	5.86 / 351.84	0.60
Human Myr-PTEN-PDZ	One phase decay, Least square fit	6.17	0.11/ 6.74	0.57
Mouse Myr-PTEN-PDZ	One phase decay, Least square fit	0.80	0.87/ 52.16	0.17
50% *w*/*v* Brain Homogenate				
Human Lau-PTEN-PDZ	One phase decay, Least square fit	1.90	0.36 / 21.87	0.88
Mouse Lau- PTEN-PDZ	One phase decay, Least square fit	3.48	0.20 / 11.95	0.84
Human Myr-PTEN-PDZ	One phase decay, Least square fit	4.20	0.16 / 9.89	0.61
Mouse Myr-PTEN-PDZ	One phase decay, Least square fit	2.71	0.25 / 15.32	0.78

The clearance of therapeutic peptides is very dependent on the intense metabolism by liver enzymes, which can degrade all the peptide that reaches the bloodstream after intravenous, or other parenteral and non-invasive (oral and nasal) routes of administration, within minutes (22). Peptides with good plasma and liver stability are more likely to remain in circulation. If the peptide permeates the BBB, higher levels will reach the brain after invasive and non-invasive administration. The mouse Myr-PTEN-PDZ demonstrated the highest liver stability (Fig. 2b and Table I).

The mouse PTEN-PDZ lipidized peptides (Myr/Lau) were more stable in murine plasma (50% *v*/*v*), exhibiting longer half-lives than the human peptides and with more than 50% of the peptide remaining after a 2-h incubation (Fig. 2c-d, Table I). These longer mouse plasma half-lives can be explained by differences in protein binding between the two species (23,24). Based on the higher lipophilicity of the myristoylated peptides, lauryl peptides are expected to bind less protein (1). Lipopeptides can permeate via both active and passive processes across the BBB. The free peptide fraction will be available for transport transcellularly, while protein binding to certain plasma proteins can induce receptor mediated uptake across the BBB. Thus, laurylated peptides are expected to be more available for transcellular transport, while more mechanistic studies are needed to elucidate the mechanism of BBB permeation and the biological effect of protein binding of these lipopeptides. Based on the above, the mouse Lau-PTEN-PDZ peptide, showing good stability in mouse plasma and a half-life of 5.9 h (Table I), would appear to be a good candidate for further development via parenteral routes. Lastly, all four peptides show good *in vitro* brain homogenate stability with the human Lau-PTEN-PDZ, and mouse Myr-PTEN-PDZ demonstrated better stability than the other peptides (Fig. 2d and Table I).

### Self-Assembly of Lipidized Peptides in Micromolar Concentrations

**Fig. 3:**
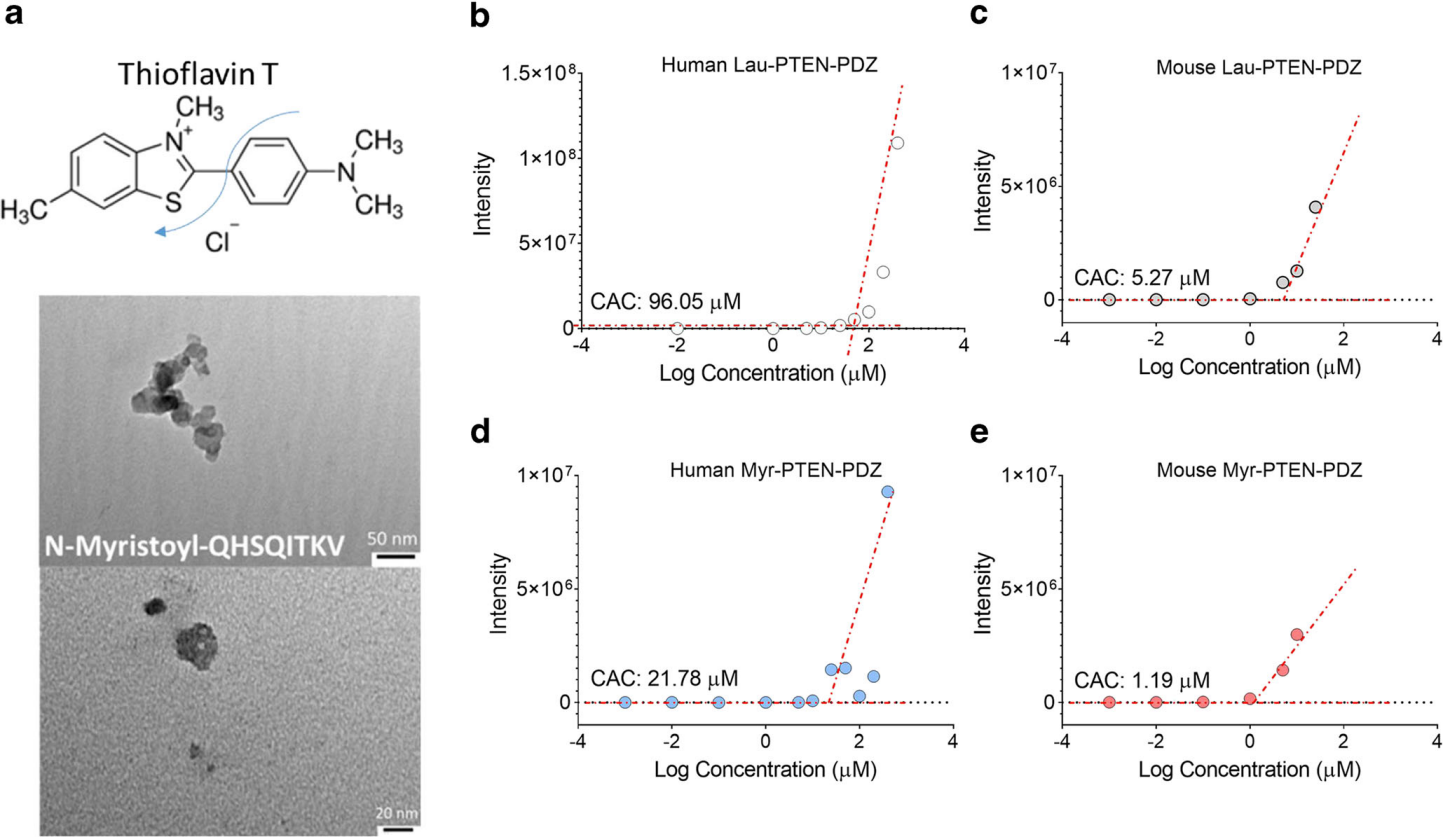
(**a**) Top: Thioflavin T structure; Bottom: TEM images of mouse Myr-PTEN-PDZ supramolecular structures (2% Uranyl acetate staining, scale bar = 50 and 20 nm). (**b**-**e**) Thioflavin T aggregation assay of peptide oligomers (**b**: Human Lau-PTEN-PDZ, **c**: Mouse Lau-PTEN-PDZ, **d**: Human Myr-PTEN-PDZ, **e**: Mouse Myr-PTEN-PDZ) (n = 3).

Although small peptides typically adopt flexible conformations in aqueous media, lipopeptides have a more stable structure due to their ability to self-assemble into oligomers or larger aggregates of varied morphologies (i.e., spherical, long-axial, tapes or ribbons). Self-assembly of peptide amphiphiles is driven by gains in entropy. Such gains are associated with the dehydration of hydrophobic moieties and the resulting hydrophobic associations as the water molecules adjacent to the hydrophobic part of the peptide are forced to assume a higher-ordered structure, and they become unable to hydrogen bond freely. This gain in entropy by the water molecules is what ultimately drives the process of self-assembly (25). These stabilized structures can be manipulated to enhance their resistance to enzymatic degradation. We used the thioflavin T assay to calculate the aggregation concentrations of the lipopeptides. Mouse peptides had close to a 4-fold lower critical aggregation concentrations (CACs), in the micromolar range (Fig. 3). As expected from the tail length and critical packing parameter (25,26), the myristoylated peptides assemble at 20-fold lower CACs than the laurylated peptides. The formation of spherical oligomers would be expected due to the use of a saturated linear hydrophobic tail less than 16 carbons in length, as shown by TEM of mouse Myr-PTEN-PDZ. The formation of small spherical oligomers might explain their enhanced stability in biological media and, to some extent, the enhanced stability of the murine PTEN-PDZ peptides.

### Cell Treated with the Mouse Myr-PTEN-PDZ Shows Cytotoxicity

**Fig. 4:**
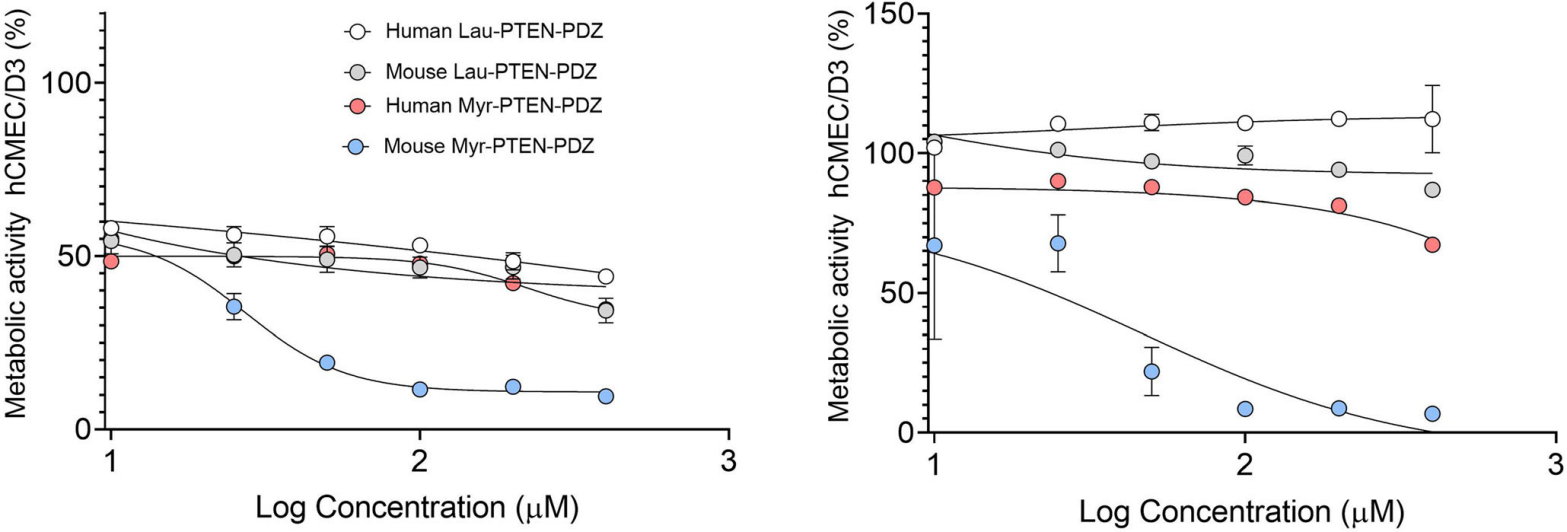
Cell metabolic activity (%) in hCMEC/D3 cells versus log concentration of the peptides after a 4-h (left)  and 24-h (right) exposure. Data are normalized as % control using GraphPad Prism 8.02 and presented as means ± SEM (*n* = 3).

The toxicity of the peptides to hCMEC/D3 cells was assessed prior to testing them in our *in vitro* BBB model to ensure that they were well tolerated at the concentrations used. The MTT assay demonstrated acute toxicity of hCMEC/D3 cells to the peptides after 4 h, yet these cells recovered at 24 h. Indeed, more than 80% of the cells remained metabolically active when exposed to the peptides at a concentration of 200 μM that was intended to be used in the BBB permeability assays (Fig. 4). However, this was not the case for the mouse Myr-PTEN-PDZ, the only peptide that did not allow the cells to recover after 24 h.

### Laurylated Peptides Are Permeable across the BBB

As the BBB protects the brain from unwanted chemicals in the blood, it may hinder the passage of therapeutic compounds to the brain (27,28). Nonetheless, lipid-mediated transport favors the passage of lipid-soluble small molecules, below a 400–600 Da threshold, across the BBB (29). Thus, in addition to selecting compounds with a strong potential for BBB transport, we employed an all human 2D *in vitro* BBB Transwell model (30,31) to test their penetration through a model of the BBB comprised by human immoralised brain capillary endothelial cells (hCMEC/D3) and SC-1800 astrocytes in the blood-to-brain direction. The passage of each molecule from the ‘blood’ compartment of a single insert across the barrier was measured at multiple time points, detecting the amount of the peptide in the ‘brain’ compartment and calculating the apparent permeability coefficient (Papp) for all the peptides.

Lauryl peptides were almost twice as permeable across the BBB than the myristoylated peptides, with the mouse Lau-PTEN-PDZ demonstrating the highest permeability (6.3 ± 1.8 × 10^−6^ cm/s, Table II). The Papp values calculated for the controls (FITC-dextran and diazepam) were similar to those reported previously (16,21,32,33), with typical values for FITC-dextran ranging between 0.2–7.3 × 10^−6^ cm s^−1^, further evidence of the validity of our model (34). Significantly, the Papp for the mouse Lau-PTEN-PDZ peptide was in the same range as that reported for brain permeable peptides like Angiopep-2 Papp, 8.69 ± 1.53 × 10^−6^ cm/s (35).

**Table II:** Permeability of the Compounds Across an *In Vitro* 2D Human BBB Model

Compound	Experiment 1	Experiment 2
	Papp_0-4h_ (×10^−6^ cm s^−1^)	Papp_0-4h_ (×10^−6^ cm s^−1^)
Human Lau-PTEN-PDZ		5.57 ± 0.29
Mouse Lau-PTEN-PDZ	6.28 ± 1.85	
Human Myr-PTEN-PDZ		3.77 ± 0.39
Mouse Myr-PTEN-PDZ		2.63 ± 0.08
FITC-Dextran (3–5 kDa)	6.91 ± 0.30	
Diazepam	19.07 ± 0.58	18.90 ± 0.86

## Discussion

We have analyzed here the possible clinical utility of four PTEN-PDZ lipopeptides by testing their stability in biological fluids, their cytotoxicity, their ability to self-assemble, and their BBB permeability. Using the mouse Myr-PTEN-PDZ as a prototype, we developed three similar lipopeptides based on the human/mouse PTEN C-terminal sequence. We set out to determine which one of these potential drugs would be the most suitable agent for preclinical development as a novel therapeutic agent to combat memory loss in AD.

Therapeutic peptides generally have poor oral bioavailability (injection is usually required), and their capacity to cross the BBB is limited, resulting in high production costs. The oral-systemic availability of a peptide (F) is determined by the fraction of the dose that is not lost to the feces or metabolized in the gut (F_F_). The fraction that escapes degradation at the wall of the gastrointestinal tract to reach the portal vein (F_G_) and that which escapes liver metabolism also determines the oral-systemic availability of peptides (F_H_), whereby F=F_F_*F_G_*F_H_ (36). Based on the amino acid sequence of the peptides we tested, they are likely to be degraded mainly by pancreatic proteases, such as trypsin (at Lys^2^) and chymotrypsin (at His^7^). A minimally invasive or oral therapy for AD could revolutionize treatment options for AD patients even though this route of administration minimizes the efficiency and potency of potential drug therapies (37). Our stability studies in a SIF that contains pancreatic proteases, among the soluble enzymes present in the gastrointestinal tract, indicate that the prototypic mouse Myr-PTEN-PDZ lipopeptide and possibly the derived human Lau-PTEN-PDZ might be suitable candidates for oral delivery. Nevertheless, the former has lower plasma stability to human Lau-PTEN-PDZ, but the latter exhibits low liver stability. Thus, it seems that another minimally invasive route, either parenteral or nasal, is likely to yield higher brain levels of these peptides and produce a better therapeutic response, with a minimal dose and lower cost.

We report here that while the mouse Myr-PTEN-PDZ peptide shows acceptable stability in most of the biological fluids (Table III), its BBB permeability is low compared to the other peptides, and it shows a high cytotoxicity rate. The human Myr-PTEN-PDZ peptide shows low stability in plasma and a relatively low BBB permeability. On the other hand, the human Lau-PTEN-PDZ shows good stability in most biological fluids, a good BBB permeability, and less cytotoxicity. The mouse Lau-PTEN-PDZ, followed by the human Lau-PTEN-PDZ peptide, represents the least toxic of the peptides for human immortalized cerebral endothelial cells, which constitute the luminal side of the BBB. The mouse Lau-PTEN-PDZ peptide shows good stability in mouse plasma, good BBB permeability, and low cytotoxicity. Moreover, these two derivatives are the leading candidates for delivery via minimally invasive routes (intramuscular, subcutaneous, and nasal) due to their enhanced permeability across a 2D human BBB model. Nevertheless, human Lau-PTEN-PDZ is not very stable in mouse plasma (50%), and therefore, it is less suitable for preclinical research. Only the mouse Lau-PTEN-PDZ has both good BBB permeability and plasma stability. At the same time, this peptide is likely to bind less protein as it is less lipophilic. A long plasma half-life is essential to elicit an effective brain response. Intravenous, subcutaneous, intramuscular, and nasal delivery are potential routes for these peptides. The increased stability of peptides in biological media can be attributed to their amphiphilic character and to their ability to self-assemble in aqueous media in micromolar concentrations. Serine endopeptidases or threonine endopeptidases are enzymes likely to be involved in the plasma, and tissue degradation of these peptides, as well as proteinase K. Previous studies showed that in terms of permeability across Caco-2 cells, lipidation with a shorter C_8_ tail is more beneficial than a C_12_ tail for amine-modified peptides (38), although we do not know if this is the case for these peptides. Even if the permeability of shorter tail lipopeptides is high, if they are not stable in the gastrointestinal tract, their oral bioavailability *in vivo* will be limited. Finally, liver stability and permeability across the BBB needs to be taken into consideration, and taking all factors together, mouse myristoylated peptides appeared to be the best candidate of the lipopeptides tested for oral development.

**Table III:** Pharmacological Profiles of the Peptide Drug Candidates

Factors	Human Lau-PTEN-PDZ	Mouse Lau- PTEN-PDZ	Human Myr-PTEN-PDZ	Mouse Myr-PTEN-PDZ
Simulated intestinal fluid stability	**+++**	**++**	**+++**	**+++++**
Liver homogenate stability	**++**	**++**	**++**	**+++++**
Plasma stability (mouse, 50% *v*/*v*)	**++**	**+++++**	**++**	**+++**
Brain homogenate stability	**+++**	**+++**	**++++**	**+++**
In vitro BBB permeability	**++++**	**++++**	**++**	**+**
Cell viability	**+++++**	**+++++**	**+++++**	**+**

The stability data so far indicate that mouse Lau-PTEN-PDZ was more stable in plasma (50% *v*/*v*), ensuring a longer half-life in circulation and allowing more of the peptide to be transported across the BBB and would make it a good candidate for parenteral (intravenous, intramuscular) administration, yet also candidates for nose-to-brain delivery. This latter route not only delivers peptides to the brain via the olfactory pathway, but it can lead to high plasma levels, mimicking the pharmacokinetic profile achieved after intravenous administration and potentially resulting in more peptide accumulating in the brain if it is brain permeable. Thus, it was critical to assess *in vitro* BBB permeability to decide which of the lipopeptides should be taken further into preclinical development. Nose-to-brain delivery strategies might be preferred to treat a chronic condition like AD, permitting patient self-administration without the need for complicated training. Advances in nasal devices and formulations will probably produce very stable formulations, and that can ensure 1–2% of the nasally administered dose reaches the brain (1,3).

The permeability of mouse/human Lau-PTEN-PDZ peptides is similar to that reported for brain permeable peptides like Angiopep-2 based on the Kunitz domain of aprotinin (Papp (cm/s) = 8.69 ± 1.53 × 0^−6^ [36]). This domain is brain permeable *in vitro* and *in vivo*, on its own, as a drug conjugate or as a peptide-functionalized nanomedicine that can be loaded with a therapeutic cargo. Current permeability values indicate that at financially viable doses, 0.2% of an intravenously injected dose can reach the brain. This value may be further enhanced if the nasal route is selected for delivery with an appropriate device (3). The nasal route by-passes the BBB and delivers peptides straight into the brain. Through this route, the peptides can elicit blood levels with clearance similar to that observed after intravenous administration. Thus, the nasal route might potentiate the accumulation of the brain permeable peptide with a long half-life in circulation, resulting in brain levels that reach 1–2% of the dose administered. The diffusion of therapeutic peptides (<10 kDa) into the extracellular brain parenchyma, such as that described here, is not restricted significantly when compared to that of larger neurotrophic factors like neurotrophins, PDGF (platelet-derived growth factor) and CNTF (ciliary neurotrophic factor) which approach the dimensions of the extracellular space (15–20 nm in diameter) (39). The clearance of the peptides in the target organ is also an essential feature, as this will permit the degradation of the therapeutic peptides into elements that are naturally present in the brain parenchyma (40).

As such, these peptides may serve as potential candidates for pharmacological interventions in conditions of Aß overload, and by saturating PTEN-PDZ interaction sites, we can effectively protect synapses against Aβ, preventing the cognitive deterioration triggered by Aβ overload.

## Conclusions

We show here that PTEN-PDZ lipopeptides may represent a feasible clinical strategy for the symptomatic treatment of AD patients. The interactions of therapeutic peptides differ from those of small molecules, and their toxicity and immunogenicity are limited, making them ideal candidates to treat brain diseases. Lipidized peptides have a longer half-life in circulation and enhanced permeability across biological barriers. Examining different analogs of PTEN-PDZ peptides, we have identified the optimal lipidic tail for stability and permeability across difficult biological barriers like the BBB, maintaining low cytotoxicity. We identified the mouse Lau-PTEN-PDZ as the right candidate for further development as a minimally invasive medicine for self-administration via the nasal, intramuscular, or subcutaneous routes in order to prevent or delay memory defects in the early phases of AD.

## Abbreviations

Aβ: Amyloid β
AD: Alzheimer’s Disease
BBB: Blood-brain barrier
hCMEC/D3: Human cerebral microvascular endothelial cells
PDZ: PSD95/Disc large/Zonula occludens-1 proteins
PTEN: Phosphatase and Tensin Homolog Deleted on Chromosome 10
SC-1800: Human astrocytes
